# Proinflammation and Hypertension: A Population-Based Study

**DOI:** 10.1155/2008/619704

**Published:** 2008-12-30

**Authors:** Mauno Vanhala, Hannu Kautiainen, Esko Kumpusalo

**Affiliations:** ^1^Unit of Family Practice, Central Hospital of Middle Finland, 40620 Jyväskylä, Finland; ^2^Department of Family Medicine, School of Public Health and Clinical Nutrition, Unit of Family Practice, Kuopio University Hospital, Kuopio University, 70211 Kuopio, Finland; ^3^Department of Rheumatology, Rheumatism Foundation Hospital, 18120 Heinola, Finland

## Abstract

There is evidence that proinflammation may be linked to the development of hypertension (HT). We examined the association of both the interleukin-1 beta (IL-1*β*) and the interleukin 1-receptor antagonist (IL-1ra) with future blood pressure (BP) and HT occurrence (BP ≥ 140/90 mmHg, or antihypertensive drug) in a population-based prospective study. Our study consisted of 396 (147 men and 249 women) middle-aged, baseline apparently healthy, normotensive subjects participating in a 6.5-year follow-up study. Subjects with high-sensitivity CRP (hs-CRP) < 10 mg/L were excluded at the initial visit. At follow-up, the occurrence of HT was 32%. The levels of baseline IL-1*β* and IL-1ra were significantly higher for subjects who developed HT during the follow-up than for those who did not (IL-1*β*; 0.67 ± 0.62 pg/mL versus 0.56 ± 0.32 pg/mL, *P* = .020 and IL-1ra; 184 ± 132 pg/mL versus 154 ± 89 pg/mL, *P* = .007). After adjustments for age, follow-up time, sex, baseline systolic BP, and BMI, our results confirm a statistically significant (*P* = .036) linear association between the quartiles of IL-1*β* and change of systolic BP during the study. After adjustments for age, follow-up time, sex, and BMI, our results also show a linear association between incident HT and the quartiles of IL-1ra. (*P* = .026). These results provide evidence that proinflammation may precede BP elevation and HT.

## 1. INTRODUCTION

There exists a growing body of evidence describing the role of cytokines in the context of hypertension (HT) [1–5]. Interleukin-1beta (IL-1*β*) is a crucial mediator of inflammatory response. Animal experiments that examined the vascular wall of hypertensive rats showed an increase in mRNA expression levels of IL-6, IL-1*β*, and TNF-alpha [6]. Previous human work has shown increased levels of IL-1*β* in the systemic circulation of patients with essential HT. Most IL-1*β* is secreted by peripheral blood monocytes instead of adipocytes in hypertensive subjects, and angiotensin II (Ang II) may be directly involved in the process of monocyte activation [7]. IL-1*β* and Ang II together may also regulate sympathetic nervous system (SNS) activity through negative feedback by modulating the expression of nitric oxide (NO) in the brain [8–10]. IL-1*β* could also be an additional risk factor for atherogenesis in hypertensive patients [11].

The production and effects of IL-1*β* are controlled at many levels, a critical one being the inhibition of its activities by the secreted form of the interleukin-1 receptor antagonist (IL-1ra) [12]. Elevated concentrations of this substance have been connected to insulin resistance and type 2 diabetes [13]. Moreover, elevated levels of IL-1ra have been found in subjects with essential HT [14]. Even if proinflammation is linked to cardiovascular disease (CVD), there currently exists no evidence showing that IL-1ra could have an independent role in the etiology of CVD [15].

IL-1*β* also stimulates the synthesis of hs-CRP and other inflammatory mediators [16–18]. It has been speculated that inflammation plays an important role in triggering vascular fibrosis, which is an important aspect of extracellular matrix remodeling in hypertension [19]. Another research has proven that CRP predicts future cardiovascular events, at least in women [20].

Baseline blood pressure (BP), obesity, use of alcohol, and lack of physical activity have traditionally been suggested as precursors of HT that works via mechanisms including proinflammation and insulin resistance [21, 22].

Despite several reliable cross-sectional studies showing the connection of IL-1*β* and IL-1ra to hypertension, researchers have not yet conclusively linked these proinflammatory markers to blood pressure in a longitudinal study. Accordingly, we examined the two aforementioned proinflammatory markers, lifestyle factors, and other traditional risk factors in a normotensive, apparently healthy population. Our goal was to understand their roles in both future BP changes and HT in follow-up examinations.

## 2. MATERIALS AND METHODS

### 2.1. Subjects

All subjects (*N* = 1294) were born in 1942, 1947, 1952, 1957, and 1962 in Pieksämäki, a town in eastern Finland. The patients were invited to participate in a general health examination in 1997-1998. A total of 923 of the 1294 subjects (71.3%) participated in an initial examination in 1997-1998, and 690 of those underwent a second checkup in 2003-2004. The mean follow-up time was 6.45 (range 5.3–7.4) years. There were no additional examinations between these two checkups. All participants gave informed written consent. The study protocol was approved by the Ethics Committee of the Kuopio University Hospital and the University of Kuopio.

All participants completed a standard questionnaire in the beginning and at the end of the study. It included questions about smoking habits, use of alcohol, and physical activity. Those who smoked daily were classified as smokers. Use of alcohol was measured using a scale with ten graded levels: 0 = never used, 1 = not used alcohol in the last year, 3 = used less than 1 unit (standard drinks)/week, 4 = 1-2 units/week, 5 = 3–5 units/week, 6 = 6–9 units/week, 7 = 10–14 units/week, 8 = 15–21 units/week, 9 = 22–28 units/week, and 10 = more than 29 units/week. A subject was classified as drinking more than the recommended 1-2 doses/day if he/she consumed 10 or more alcohol units/week. Physical activity was measured using a scale that comprised six graded levels. The patients were asked to define physical exercise as sufficiently strenuous exertion that results in perspiration and breathlessness. 1 = daily exercise, 2 = three or more times/week, 3 = twice/week, 4 = about once/week, 5 = once/month, and 6 = less than once/month. A subject was classified as physically active if he/she exercised three or more times per week.

All patients with hs-CRP > 10 mg/L at the initial examination were excluded due to the fact that they might have had an active infection. Since certain cardiovascular drugs can affect BP and cytokines, all patients taking medication for another cardiovascular disease were excluded in the beginning of the study.

### 2.2. Clinical and laboratory methods

Height and weight were measured to the nearest 0.5 cm and 0.1 kg, respectively. Body mass index (BMI) was calculated as weight (kg) divided by height (m) squared. The waist was measured to the nearest 1.0 cm at the midpoint between the lateral iliac crest and the lowest rib. Trained nurses measured BP twice with subjects in a seated position using a mercury sphygmomanometer. The mean of two measurements was used for subsequent statistical analyses. Measurements were performed at the initial and final visits. Hypertension was determined to be present if subjects had a BP ≥ 140/90 mmHg or if subjects were taking antihypertensive medication [23].

Fasting blood samples were drawn after 12 hours of fasting. C-reactive protein (CRP) was measured with an Immulite (R) and a DPC high-sensitivity CRP assay (hs-CRP). Plasma concentrations of IL-*β* and IL-1ra were measured using assay kits from R&D Systems (Minneapolis, Minn, USA; for IL-1*β*, resolution power was 0.125–8 pg/mL and sensitivity 0.1 pg/mL; for IL-1ra, resolution power was 46.9–3000 pg/mL and sensitivity 22 pg/mL). Cytokines were measured during the initial examination.

### 2.3. Statistical analysis

The results were expressed as means, standard deviations (SDs), ranges, and 95 percent confidence intervals (95% CI). IL-1*β* and IL-1ra exhibited skewed distributions and were, therefore, logarithmically transformed for statistical analysis. The *t*-test and chi-squared test were used to derive statistical comparisons between genders or between groups of hypertensive and normotensive subjects in respect of baseline characteristics and other subgroup parameters. The hypotheses of linearity between quartiles of plasma cytokine levels and changes in systolic BP or occurrence of HT were analyzed with two general linear models known as the analysis of covariance (ANCOVA) and with logistical models. All models were adjusted for gender, age, baseline BMI, follow-up time, and baseline systolic BP when appropriate. All statistical tests were two sided, withan alpha-level of 0.05.

## 3. RESULTS

Of the 233 nonparticipant subjects, 48% (*n* = 112) were men and 52% (*n* = 121) were women. Our data shows no statistical difference in BP, BMI, age, or lifestyle factors in nonparticipants as compared with the participants. Of the 690 participants, 406 subjects (59%) had a BP < 140/90 mmHg and were not undergoing antihypertensive drug treatment at the time of the baseline checkup. Six of these patients had hs-CRP > 10 mg/L, and four subjects were taking a certain drug for another CVD; all of such participants were excluded. Thus, the final study population consisted of 396 apparently healthy subjects (147 men and 249 women).

At the initial checkup, mean systolic BP was 126 ± 8 mmHg in male patients and 122 ± 10 mmHg in female subjects (*P* between sexes < .001). Mean diastolic BP was, respectively, 77 ± 6 mmHg and 75 ± 7 mmHg (*P* < .001). BMI in men was higher than in women (25.9 ± 3.1 kg/m^2^ versus 25.1 ± 3.6 kg/m^2^, *P* between sexes = .029). Twelve percent of men consumed 10 or more units of alcohol each week, and this was statistically significantly higher than the 2% of women who reported the same alcohol consumption (*P* between sexes < .001). There was no sex-related difference in physical activity. The percentages of physically active subjects were 30% in men and 28% in women.

The levels of cytokines recorded at the baseline checkup were statistically similar for both sexes. The level of IL-1*β* was 0.59 pg/mL ± 0.43 pg/mL in men and 0.58 pg/mL ± 0.43 pg/mL in women. The corresponding figures for IL-1ra were 157 pg/mL ± 92 pg/mL in men and 168 pg/mL ± 102 pg/mL in women. There was no statistically significant correlation between these two cytokines.

Between the initial and follow-up examinations, smoking decreased from 28% to 23% in men and from 21% to 17% in women, and consumption of 10 or more alcohol units/week increased from 12% to 18% in men and from 2% to 4% in women. The percentage of physically active subjects did not change.

Between examinations, the systolic BP increased 9 ± 12 mmHg and the diastolic BP increased 4 ± 8 mmHg. In total, 128 (32.3%) of the 396 subjects, 59 men (41%) and 69 women (27%, *P* = .005 between sexes), developed HT. Five of those 59 men and 14 of those 69 women were prescribed antihypertensive medication between the initial and the subsequent visit.

At the end of the follow-up, six (4%) men and seven (3%) women were receiving drug treatment for another cardiovascular condition. The use of antilipolytic medication increased among the men from four (3%) to 28 (19%) users, and in women from two (1%) to 27 (11%) users over the duration of our study. In those subjects treated with lipid-lowering drugs at the end of the study, the mean systolic BP by the end of our study was 138 ± 16 mmHg, as compared to 131 ± 14 mmHg in the nontreated subjects (*P* < .001 between groups). The corresponding figures for diastolic BP were 82 ± 9 mmHg and 80 ± 9 mmHg, *P* = .060.

Table 1 shows that the mean level of baseline IL-1*β* was significantly higher among those subjects who had developed hypertension between the initial and the subsequent visits as compared to those subjects who were normotensive at the time of the second checkup (0.56 pg/mL versus 0.67 pg/mL, *P* = .020). The same phenomenon was present in the levels of IL-1ra (154 pg/mL versus 184 pg/mL, *P* = .007).

**Figure 1:**
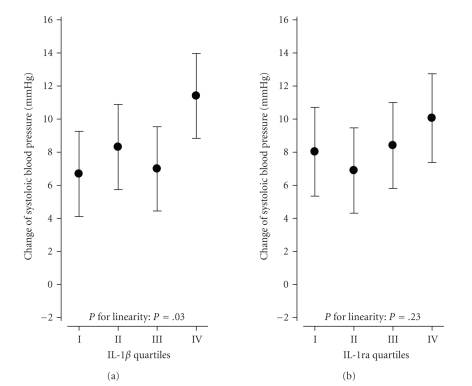
Change of systolic blood pressure (mmHg) by IL-1*β* and IL-1ra quartiles among 377 baseline normotensive subjects without antihypertensive drug treatment at the end of the 6.5-year prospective study. Adjusted for gender, age, baseline BMI, baseline systolic blood pressure, and follow-up time.

The systolic BP increased 6 ± 14 mmHg from the lowest to the highest quartile of IL-1*β*. This difference was statistically significant (*P* = .008). The BP values sorted by quartile of IL-1*β* concentration were 129 ± 13 mmHg, 132 ± 16 mmHg, 130 ± 14 mmHg, and 135 ± 15 mmHg (*P* for linearity = .019). Figure 1 shows that after adjusting gender, age, follow-up time, BMI, and baseline systolic BP, a statistically significant (*P* = .036) linear association was evidenced between the quartiles of IL-1*β* and the change in the systolic BP during the follow-up. Further adjustment for smoking, use of alcohol, and physical exercise at baseline did not change the results. Taking into account lipid-lowering medication or drugs for other cardiovascular disease did not change these results.

**Figure 2:**
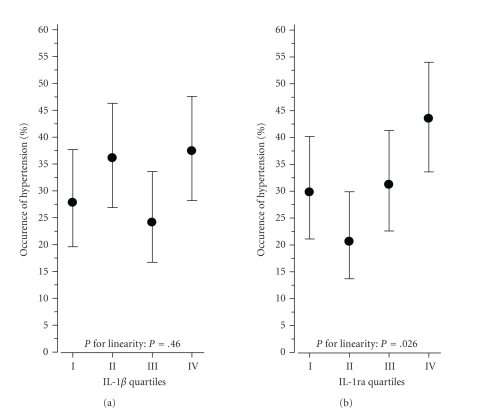
Occurrence of hypertension (RR ≥ 140/90 mmHg or drug treatment for hypertension) over a 6.5-year timeframe according to quartiles of baseline IL-1*β* and IL-1ra. Adjusted for gender, age, baseline BMI, and follow-up time.

The prevalence rates of incident HT, from the lowest to the highest quartile of IL-1ra, were as follows: 26.5%, 23.2%, 34%, and 45.5% (crude *P* = .003 for linearity). The increase in HT occurrence from the lowest to the highest quartile of IL-1ra was 19% (*P* = .05 after adjustments for gender, age, baseline BMI, and follow-up time). Table 2 and Figure 2 show that there was also a linear association between HT incidence and quartiles of IL-1ra (*P* = .026) after adjustments as previously described. Further adjustment for smoking, use of alcohol, and physical exercise at baseline did not change the results. Taking into account lipid-lowering medication or drugs for other cardiovascular disease did not change these results.

## 4. DISCUSSION

To the best of our knowledge, this study is the first prospective, population-based study providing evidence that high levels of proinflammatory cytokine IL-1*β* precede future changes in systolic BP and in levels of its specific antagonist IL-1ra in the context of HT. These associations were present after adjustments for sex, age, BMI, and follow-up time; they also remained for analyses that only examined BP from baseline.

Earlier findings suggest that the pressor effect of IL-1*β* is mediated by AT1 receptors and depends on the stimulation and the release of Angiotensin II (Ang II). [20, 21] Ang II raises BP via its peripheral and central effects. In the brain, ANG II downregulates neuronal NOS (nNOS) and leads to the decrease of NO production and the increase of SNS activity [8, 9, 18, 24]. There are also observations that IL-1*β* downregulates SNS activity by increased local expression of NOS mRNA as well as that Ang II-mediated increases in arterial BP can be caused by inhibition of the expression of IL-1*β* and nNOS at the brain level [10]. ANG II and IL-1*β* may thus act in a negative feedback loop in the central BP control system [8]. It seems possible that central IL-1*β* is also involved in stress-induced HT [25].

The unbalanced production of IL-1*β*, and of its natural specific inhibitor IL-1ra, plays an important role in chronic/sterile inflammation [12]. IL-1ra is an acute phase reactant for IL-1*β*, since it reflects inflammatory reactions, acts in an antagonistic manner, and serves as a natural compensatory mechanism for the IL-1-induced disease process. IL-1ra concentrations increase during both insulin resistance and the metabolic syndrome [13]. A human experiment has shown that IL-1*β* induces the secretion of IL-1ra, and that induction of IL-1*β* precedes secretion of IL-1ra.

In our study, IL-1*β* is independently associated (even after taking into account age, sex, baseline BMI, and certain lifestyle factors) with changes in systolic BP that may reflect an incipient change in arterial stiffness. High levels of IL-1ra seem to be linked to occurrence of HT, and this can most likely be explained by the fact that those subjects already in the initial examination exhibited elevated BP subsequently developed stable HT (defined as ≥140/90 mmHg and/or prescribed antihypertensive drugs). These findings are in line with previous data suggesting that proinflammation precedes HT and that it may be a trigger for arterial wall stiffness [20].

Lipid-lowering medications and some drugs used to treat cardiovascular diseases other than HT may have BP lowering effects [26]. Evidence also suggests that lipid-lowering medications can affect the levels of cytokines [27]. Therefore, we excluded subjects who were using this kind of medication at the beginning of the study. Previous work has shown that lifestyle factors like exercise, smoking, and consumption of alcohol can affect both BP and HT [21, 22]. Therefore, we used the baseline information to correct our analyses for these potential confounding factors.

During the course of the study, 59 men (41%) and 69 women (27%, *P* = .005 between sexes) developed HT. This is consistent with both earlier results [22] and the knowledge that middle-aged men are at greater risk for all cardiovascular diseases than women. Our study also showed that lipid-lowering medications were prescribed more frequently for men; this finding is also consistent with the present recommendations [26]. Antilipolytic medication use increased during the study, especially among those subjects who developed HT. Therefore, the increase occurred in subjects with a high risk for having HT and dyslipidemia simultaneously.

One strength of this study lies in its population-based, prospective design, which includes both men and women and evaluates many traditional risk factors simultaneously. We also took into account the possibility that lifestyle factors and some drug treatments may have an effect on the results by correcting our analysis for these confounding factors. Lifestyle factors are very difficult to evaluate accurately [28], and one shortcoming in our study is that these factors were self-reported. We also did not measure sympathetic nervous activity or endothelial function at the initial visit or levels of cytokines during the subjects' subsequent visit. Future studies should evaluate the possible association of sympathetic overflow and cytokines with respect to HT.

We conclude that our report provides important evidence regarding the relationship between cytokines and future BP or HT. This work shows that IL-1*β* and IL-1ra are markers for future BP and HT, and thus for prehypertension, in the normotensive, apparently healthy population. We suggest that both IL-1*β* and IL-1ra reflect the same phenomenon, namely, vascular inflammation and arterial stiffness. The clinical importance of this study lies in the fact that both Il-1*β* and IL-1ra are linked to increased cardiovascular risk in hypertensive subjects. This increased risk may reflect the presence of extended-duration proinflammatory vasculature stress.

## 5. SUMMARY

What is known about this topic?

- There is evidence that proinflammation may be linked to the development of hypertension.
- Increased levels of IL-1*β* and IL-1ra have been shown to be linked with essential hypertension in cross-sectional studies.

What this study adds?

- This longitudinal study of apparently healthy middle-aged men and women shows that high levels of IL-1*β* and IL-1ra are risk factors for subsequent hypertension.

## Figures and Tables

**Table 1:** Baseline characteristics of 396 baseline normotensive subjects according to hypertension status at the end of the follow-up period. HT−: normotensive at the end of study, HT+: hypertensive at the end of study (blood pressure ≥140/90 mmHg and/or antihypertensive drug use), and CVD: cardiovascular disease.

	HT−	HT+	*P*
	*n* = 268	*n* = 128	
Number of female = 249/396 (63%)	180 (67%)	69 (54%)	—
Age (years)	50.6 ± 6.1	53.0 ± 51	<.001
Follow-up time (years)	6.4 ± 0.4	6.4 ± 0.4	.091
BMI (kg/m^2^)	25.1 ± 3.6	26.0 ± 3.6	11
IL-1*β* (pg/mL)	0.56 ± 0.32	0.67 ± 0.62	.020
IL-1ra (pg/mL)	154 ± 89	184 ± 132	7
Lipid-lowering drug at the beginning of the study	1%	3%	.091
Lipid-lowering drug at the end of the study	9%	26%	<.001
Drug for some other CVD at the end of the study	3%	6%	.398
Smoking	30%	26%	.807
Alcohol > 10 units/week%	5%	6%	.807
Physically active	25%	33%	.086

**Table 2:** Odds ratios of hypertension (≥140/≥ 90 mmHg or drug treatment for hypertension) according to quartiles of plasma levels of baseline IL-1*β* and IL-1ra.

Variable	Quartile of plasma level				*P* for linearity*
	I	II	III	IV	
IL-1*β*					
Median (range), pg/mL	0.25 (0.088–0.378)	0.48 (0.379–0.566)	0.63 (0.567–0.696)	0.82 (0.700–5.540)	
Odds ratio (95% CI)	1.00	1.47 (0.79 to 2.73)	0.83 (0.43 to 1.58)	1.55 (0.84 to 2.89)	.46

IL-1ra					
Median (range), pg/mL	84 (46–98)	117 (99–139)	163 (140–192)	244 (193–1134)	
Odds ratio (95% CI)	1.00	0.61 (0.31 to 1.20)	1.07 (0.56 to 2.04)	1.82 (0.95 to 3.46)	.026

*P-values were calculated by using a logistic model, adjusted for gender, age, follow-up time, and baseline body mass index.
